# The exostosin family of glycosyltransferases: mRNA expression profiles and heparan sulphate structure in human breast carcinoma cell lines

**DOI:** 10.1042/BSR20180770

**Published:** 2018-08-31

**Authors:** Lawrence F. Sembajwe, Kirankumar Katta, Mona Grønning, Marion Kusche-Gullberg

**Affiliations:** Department of Biomedicine, University of Bergen, Bergen NO-5009, Norway

**Keywords:** biosynthesis, breast carcinoma cells, exostosins, gene expression, Heparan sulfate

## Abstract

Breast cancer remains a leading cause of cancer-related mortality in women. In recent years, regulation of genes involved in heparan sulphate (HS) biosynthesis have received increased interest as regulators of breast cancer cell adhesion and invasion. The exostosin (EXT) proteins are glycosyltransferases involved in elongation of HS, a regulator of intracellular signaling, cell–cell interactions, and tissue morphogenesis. The EXT family contains five members: EXT1, EXT2, and three EXT-like (EXTL) members: EXTL1, EXTL2, and EXTL3. While the expression levels of these enzymes change in tumor cells, little is known how this changes the structure and function of HS. In the present study, we investigated gene expression profiles of the EXT family members, their glycosyltransferase activities and HS structure in the estrogen receptor (ER), and progesterone receptor (PR) positive MCF7 cells, and the ER, PR, and human epidermal growth factor receptor-2 (HER2) negative MDA-MB-231 and HCC38 epithelial breast carcinoma cell lines. The gene expression profiles for MDA-MB-231 and HCC38 cells were very similar. In both cell lines *EXTL2* was found to be up-regulated whereas *EXT2* was down-regulated. Interestingly, despite having similar expression of HS elongation enzymes the two cell lines synthesized HS chains of significantly different lengths. Furthermore, both MDA-MB-231 and HCC38 exhibited markedly decreased levels of HS 6-O-sulphated disaccharides. Although the gene expression profiles of the elongation enzymes did not correlate with the length of HS chains, our results indicated specific differences in EXT enzyme levels and HS fine structure characteristic of the carcinogenic properties of the breast carcinoma cells.

## Introduction

Heparan sulphate (HS) proteoglycans (PGs) (HSPGs) are composed of HS polysaccharide chains attached to a core protein. HSPGs are ubiquitously present on the cell surface and in extracellular matrix (ECM) and act as co-receptors for many signaling molecules, such as growth factors and cytokines. The diverse and fundamental roles of HSPGs in development and homeostasis are reflected by the occurrence of HSPGs throughout the evolutionary system, from Cnidaria onward [1,2]. Furthermore, complete lack of HS results in early embryonic lethality in mouse models [3–5]. In addition, there are clearly strong relationships between HS and cancer. Protein interactions during cancer progression depend on the structural properties of HS, both on tumor cells and in the tumor stroma. Cancer cells alter their cell surface HS profile, by differential expression of particular PG protein cores, and/or by altering the fine structure of HS chains on a given PG [6–8]. Increased, as well as decreased, mRNA levels of HS biosynthesis enzymes have been reported in, for example colorectal cancer [9,10], breast and prostate cancer [10–12], and glioma [13].

The exostosin (EXT) enzymes take part in HS biosynthesis where they have a key role in generating the HS polysaccharide backbone, composed of [glucuronic acid-N-acetylglucosamine]_*n*_ [14]. The human EXT family consists of five members: EXT1, EXT2, and three EXT-like proteins (EXTL1, EXTL2, and EXTL3) [14]. EXT1, EXT2, and EXTL3 are necessary for HS chain formation [15,16] and complete loss of HS chains caused by deletion of *Ext1, Ext2*, or *Extl3* in mouse models is incompatible with life [3–5]. The functions of EXTL1- and EXTL2-proteins in HS biosynthesis are unclear. *Extl2*-deficient mice are fertile and have HS chains [17]. No *Extl1*-deficient mouse strain has been reported so far. Mutations in either *EXT1* or *EXT2* cause the human disorder hereditary multiple osteochondroma (MO), an autosomal dominant disorder characterized by bone deformities and cartilage-capped bony outgrowths, osteochondromas, at the ends of the long bones [18]. The most serious complication is malignant degeneration to chondrosarcomas, which occurs in 2–5% of the MO patients. Epigenetic inactivation of *EXT1* by promoter hypermethylation is commonly found in leukemia and non-melanoma skin cancer cells [19] and of *EXTL3* in mucinous colorectal cancers [20]. These findings suggest that alterations in these genes are involved in the disorders, since re-introduction of *EXT1* and *EXTL3*, respectively, into cancer cells induced a tumor-suppressive effect. In contrast, high expression of *EXT1* in patients with multiple myeloma is associated with a poor prognosis [21].

Increased *EXT1* mRNA expression has been found in estrogen receptor (ER)-negative breast tumors [22], whereas gene expression profiling of human breast tumor samples indicates that diminished expression of *EXT1* in early-stage ER positive lymph-node negative breast cancer patients might predict an increased risk for metastasis [23]. Furthermore, overexpression of *EXT1* in a non-tumorigenic epithelial cell line transformed these cells to a more malignant phenotype, whereas siRNA mediated down-regulation of *EXT1* in and ER-positive breast cancer cell line reduced cancer stem cell features and sensitized the cells to the chemotherapy drug, doxorubicin [24]. There is very little information on the role of the other EXTs in breast cancer. *EXT2* mutations have been identified in breast carcinoma patients [25] but if the mutations influence HS structure and function, is not known. Although alterations in the expression levels of the *EXTs* clearly affect cancer cell progression, nothing is known how these changes affect HS chain elongation, and fine structure that ultimately determines the biological effect of the enzymatic activity of the EXTs.

Functionally important processes during development and homeostasis depend on the fine structure of HS chains that provide binding sites for proteins. Although HS synthesis is not a template-driven process, the fine structure of HS chains appears to be strictly regulated in tissues [26,27], cells [28], during development [29], ageing [30], and in certain pathological conditions ([31] and references therein)*.* During the past years, the mRNA levels of HS biosynthesis enzymes (including members of the EXT family) have been determined in different tumors, and the authors have ascribed these changes to be reflected in HS structure and function [10,13]. However, the biosynthesis of HS is regulated through mechanisms that remain poorly understood and from RNA data alone, it is difficult to evaluate HSPG structure and function. Unlike DNA, RNA, and protein synthesis, the formation of HS chains is not template driven. Instead, it depends on the organization and substrate specificity of the HS biosynthetic enzymes (25 different enzymes), the availability of precursor molecules (UDP-sugars and sulphate donor) and the rate of flow through the Golgi apparatus [32,33]. In this study, we investigated the correlation between mRNA expression of EXT family members and the HS structure in breast carcinoma cell lines. We demonstrated significant changes in gene expression profiles, HS structure and enzyme activity in the epithelial breast carcinoma cells as compared with the non-tumorigenic MCF10A epithelial cells.

## Experimental

### Cell culture

All reagents used in the experiments were of laboratory grade quality. The human cell lines; MCF10A, a non-tumorigenic human mammary gland epithelial cell line; MCF7 mammary gland epithelial, luminal adenocarcinoma, ER, and progesterone receptor (PR) positive; MDA-MB-231 mammary gland epithelial basal adenocarcinoma, triple-negative for ER, PR, and human epidermal growth factor receptor-2 (HER2); and HCC38 mammary gland epithelial, ductal carcinoma, triple-negative for ER, PR, and HER2 receptors were obtained from ATCC or kindly provided by Professor Susan Fisher, University of California San Francisco. The features of the cell lines used are based on characterization in [34] and are listed in Table 1. MDA-MB231 and MCF7 were cultured in Dulbecco’s modified Eagle medium (DMEM), HCC38 in Roswell Park Memorial Institute (RPMI) medium (both from Gibco Life Technologies), supplemented with 10% FBS (Gibco Life Technologies) and a combination of 1% penicillin and streptomycin (PEST, Sigma). MCF10 was cultured in DMEM-F12 medium supplemented with cholera toxin (100 ng/ml), hydroxycortisone (0.5 µg/ml), epidermal growth factor (EGF, 20 ng/ml), insulin (10 µg/ml), 1% PEST, and 5% horse serum (all from Sigma). All cell lines were cultured at 37°C in 5% CO_2_.

**Table 1:** Cell lines characteristics

Cell line^1^	Tumor subtype	ER^2^	PR^3^	HER2 overexpression^4^	Tumor type
MCF10A	Basal	No	No	No	Non-tumorigenic
MCF7	Luminal	Yes	Yes	No	Adenocarcinoma
MDA-MB-231	Basal	No	No	No	Adenocarcinoma
HCC38	Basal	No	No	No	Ductal carcinoma

The triple-negative cell lines are defined as invasive breast cancers lacking ER and PR expression and HER2 overexpression or *HER2* gene amplification.

1 Table assembled with data from [34].

2 ER positive.

3 PR positive.

4 HER2 overexpression or gene amplification.

### Quantitative real-time PCR

Total RNA was extracted from the cells using the RNeasy mini prep kit (Qiagen). The cDNA was generated by reverse transcription using aliquots of 1 µg of total RNA with random primers (iScript cDNA Synthesis Kit, Bio-Rad) according to the manufacturer’s instructions. Quantitation of the mRNA expression profiles was done using iQ SYBR Green Supermix (Bio-Rad) in a Light-Cycler 480 (Roche Applied Sciences). Data were normalized to reference genes, hypoxanthine guanine phosphoribosyl transferase (*HPRT*) and RNA polymerase II subunit F (*POLR2F*). The primers used (Supplementary Table S1) were chosen using Primer Bank [35] or Primer BLAST – NCBI. Each primer/cDNA set was performed in triplicate and the expression level of the *EXT* mRNA was normalized to that of the reference gene mRNA level. The relative expression levels were calculated using the 2^−ΔΔ*C*^_T_ method [36].

### Western blotting analysis

Protein samples from the various cell extracts were separated on an SDS/PAGE (10% gel) and electrotransferred to a nitrocellulose membrane (Amersham Biosciences). The membrane was blocked with 5% low-fat milk in TBS-Tween buffer for 1 h, followed by incubation with a rabbit polyclonal anti-heparanase (anti-HPSE) antibody (1:1000, #APP60766, Aviva System Biology). After washing in 0.5% TBS-Tween, the membrane was incubated with anti-rabbit horseradish peroxidase conjugated (HRP) antibody (GE Healthcare), diluted 1:5000 and developed using ECL reagent (Pierce), and imaged with a ChemiDoc XRS imaging system (Bio-Rad). After stripping, the blot was stained with a mouse monoclonal antibody to β-actin (1:5000, #A544, Sigma–Aldrich). Precision plus protein control (Bio-Rad) was used as molecular weight marker.

### Glycosyltransferase assay

Crude cellular protein preparations were incubated with 0.125 µCi of ^3^H-labeled UDP-GlcNAc (62.5 µCi/µmol; prepared by mixing radiolabeled and unlabeled UDP-sugars) and 45 µg of [GlcA-GlcNAc]*_n_* oligosaccharide acceptor at 37°C overnight. Oligosaccharide acceptors were generated from K5 polysaccharide, as described [37]. Labeled products were isolated by gel chromatography and quantitated by scintillation counting.

### Flow cytometry

Cells were harvested using enzyme-free dissociation buffer (Gibco/Invitrogen Corporation) and washed twice in cold PBS. An aliquot of the cells was resuspended in 1 ml of PBS containing 3% FBS, and kept on ice throughout the entire procedure. The cells were incubated with the primary monoclonal anti-HS antibody10E4 (1:50) (from Seikagaku Corp.) for 30 min. After washing three times with PBS, the cells were incubated with the secondary antibody, allophycocyanin (APC) conjugated goat anti-mouse IgG (1:75) (Jackson Immunoresearch Laboratories, Inc.). Cells incubated with the secondary antibodies alone served as negative controls. Fluorescence was measured using an Accuri-6 flow cytometer (BD Biosciences) and analyzed with the FlowJo software (TreeStar, Inc.).

### Metabolic labeling, isolation, and analysis of HS structure

Subconfluent cell cultures were cultured with 200 µCi/ml Na_2_^35^SO_4_ (PerkinElmer) for 24 h. After 24 h, the culture medium was removed and frozen. Free glycosaminoglycan chains were isolated from the trypsin fraction (derived from cell surface/matrix proteoglycans) or from solubilized cell fractions as described [38]). Galactosaminoglycans were digested with chondroitinase ABC (Seikagaku) in 50 mM Tris/HCl pH 8.0, 30 mM Na-acetate, and 0.1 mg/ml BSA, and HS chain length was analyzed by gel chromatography on a Superose 6 HR10/30 column (Amersham Biosciences) eluted with 0.5 M NH_4_HCO_3_. Calibration of the Superose 6 column and peak elution volumes were as in [37,38] and are indicated in the chromatogram.

For disaccharide analyses, labeled HS chains were depolymerized to disaccharides by treatment with nitrous acid at pH 1.5 (which cleaves the glucosaminidic linkage at GlcNS units) yielding disaccharides from contiguous N-sulphated domains followed by reduction with NaBH_4_. Disaccharides were isolated by gel chromatography on a column (1 cm × 180 cm) of Sephadex G-15 superfine in 0.2 M NH_4_HCO_3_, and desalted by lyophilization. Labeled disaccharides were analyzed by anion-exchange HPLC using a Whatman Partisil 10-SAX column eluted with aqueous KH_2_PO_4_ of stepwise increasing concentrations at a rate of 1 ml/min. Mono-O-sulphated disaccharides were eluted with 0.026 M and di-O-sulphated disaccharides with 0.15 M KH_2_PO_4_. Peak elution volumes were assessed by calibration of the SAX-column using defined ^3^H-labeled standard disaccharides [39].

### Statistical analysis

Statistical significance was determined using the Student’s *t* test with unpaired samples. *P*<0.05 was considered statistically significant. Graphs and statistical analysis were done with GraphPad Prism v.6 (GraphPad Software, Inc., La Jolla, CA).

## Results

Previous experiments in our group have demonstrated that very low levels of EXT1 or EXT2 result in very short HS chains [37,39]. The reduction in HS chain length in turn greatly affects growth factor signaling in fibroblasts [40], tumor cell proliferation, and gene expression in a 3D hetero-spheroid model composed of fibroblasts and tumor cells [41,42]. Several publications have described global changes in mRNA levels of HS biosynthetic enzymes in different tumors and cancer cell lines. The present work was undertaken to define the role of mRNA expression levels of the EXT family members on HS structure in cancer cells by analyzing one non-tumorigenic mammary gland epithelial and three breast carcinoma cell lines (Table 1).

### Expression levels

**Figure 1:**
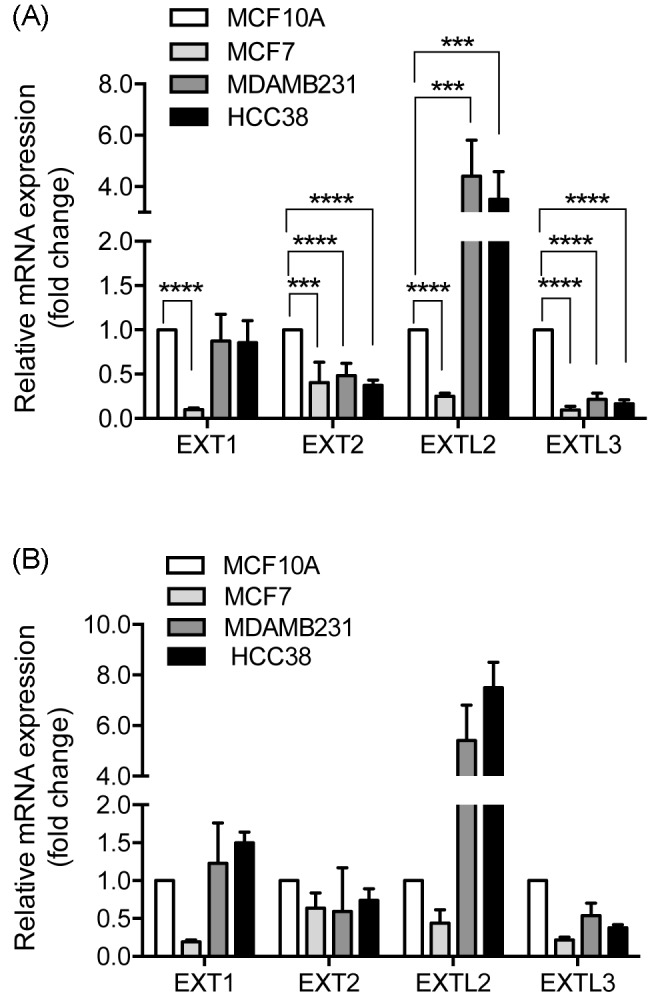
Expression levels of *EXT* gene family members Relative mRNA levels were determined by real-time PCR and normalized to (**A**) HPRT and (**B**) POLR2F. mRNA levels of MCF7, MDA-MB-231, and HCC38 were expressed relative to the non-tumorigenic MCF10A expression that was set to 1. The error bars in (A) represent the mean ± S.D. from three independent analyses performed in duplicate or triplicate. The error bars in (B) represent average values from two independent analyses performed in duplicate. EXTL1 was not detected in any of the analyzed cell lines: ***P<0.001; ****P<0.0001.

We initially examined the mRNA expression of *EXT1, EXT2, EXTL1, EXTL2*, and *EXTL3* in MCF10A, MCF7, MDA-MB-231, and HCC38 cell lines using (qRT-PCR). The results revealed that the expression of *EXT1* was significantly decreased in MCF7 cells compared with the non-tumorigenic MCF10A cells, while no significant difference was observed between the two triple-negative cell lines, MDA-MB-231 and HCC38, and the ER- and PR-positive MCF10A cells. The expression of *EXT2* and *EXTL3* was reduced in all cancer cell lines whereas *EXTL2* was down-regulated in MCF7 cells but highly up-regulated in MDA-MB-231, and HCC38. *EXTL1* was not detected in any of the cell lines (with threshold cycle (*C*_t_) vaIues ranging from 37 to 40 or blank results). Interestingly, the gene expression profiles of the two triple-negative cell lines MDA-MB-231 and HCC38 where very similar and different from the expression profile of the hormone-responsive MCF7 cell line (Figure 1).

Next we aimed to corroborate our findings from qRT-PCR analyses by determining protein levels of the EXT family members by Western blotting. We used several different antibodies, some of which were chosen from papers showing Western blots detecting the EXT family of protein. In our hands none of the antibodies recognized endogenous EXT/L proteins isolated from MCF10A cells or the tumor cell lines. Some of the tested antibodies showed unspecific binding and some recognized the overexpressed EXT family member (Supplementary Figure S1).

### HS surface expression

**Figure 2:**
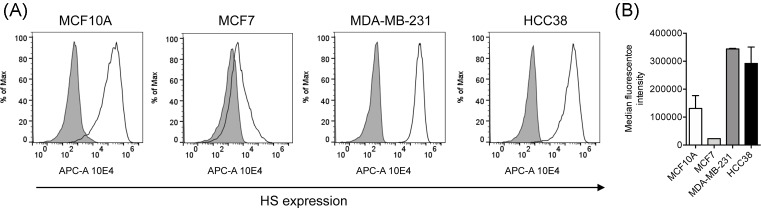
Cell surface expression of HS (**A**) Representative flow cytometry fluorescence histograms of 10E4 antibody binding to MCF10A, MCF7, MDA-MB-231, and HCC38 cells (unfilled black curves). Controls represent cells treated with the secondary antibody only (gray profiles). (**B**) Quantitated median fluorescence intensity values are shown as average ± S.D. of two separate analyses. Abbreviation: APC-A, allophycocyanin-A.

To study the relative amounts of cell surface HS expressed by the different cell types we used flow cytometry to quantitate the binding of the 10E4 antibody that is commonly used to trace HSPG. The 10E4 antibody recognizes HS domains containing both N-acetylated and N-sulphated disaccharide units [43,44]. The 10E4 antibody showed a markedly reduced binding to MCF7 cells relative to the other three cell lines (Figure 2), thus, indicating that MCF7 has less HS or a different pattern compared with the other cell lines.

To further study cell surface HS, the different breast carcinoma cells and the non-tumorigenic MCF-10A cell lines were metabolically labeled with ^35^S-sulphate. Labeled glycosaminoglycans were separately isolated from the cell surface/ECM, and the cell layer and quantitated*.* No major differences were noted in the yields of ^35^S-labeled glycosaminoglycans (calculated as cpm/mg cellular protein) between MCF10A, MCF7, MDA-MB-231, and HCC38. The relative proportions of labeled glycosaminoglycans (chondroitin sulphate (CS) compared with HS) revealed that the two triple-negative cell lines, MDA-MB-231 and HCC38, had the highest relative proportion of HS (Table 2). Thus, our data might suggest that the malignant status of the tumor cells may correlate with a shift from CS to HS synthesis.

**Table 2:** Glycosaminoglycan synthesis and disaccharide composition of labeled HS isolated from MCF10A, MCF7, MDA-MB-231, and HCC38 cells

Cell type	^35^S-labeled glycosaminoglycans	
	HS^1^ %	CS^2^ %
MCF10A	58 ± 1	42 ± 1
MCF7	56 ± 10	44 ± 10
MDA-MB-231	63 ± 10	37 ± 10
HCC38	69 ± 5	31 ± 10

Labeled glycosaminoglycans were extracted from the cell surface and quantitated. The percentage values of HS and CS are given as means from three independent ^35^S-labeling experiments ± mean deviation.

1 Material resistant to chondroitinase ABC.

2 Material susceptible to chondroitinase ABC.

**Figure 3:**
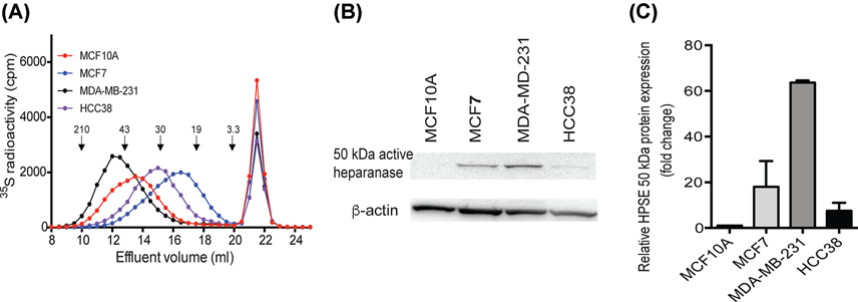
Molecular analysis of HS chains (**A**) ^35^S-labeled glycosaminoglycans were purified from the surface/ECM of MCF10A, MCF7, MDA-MB-231, and HCC38 cells as described in ‘Experimental’ section. The resulting labeled polysaccharides were digested with chondroitinase ABC and subjected to chromatography on a Superose 6 column. The retarded components eluted at approximately 21–23 ml, correspond to materials that were degraded by chondroitinase ABC. The elution positions of molecular weight standards are indicated by arrows, as in [37,38]. (**B**) Western blot analysis of HPSE protein expression in cell lysates of the different cell lines as indicated. The image shows one representative result out of two independent experiments. (**C**) Quantitation of the active HPSE 50-kDa band. Expression was normalized to β-actin and the expression in breast cancer cell lines was expressed relative to the non-tumorigenic MCF10A expression that was set to 1. Values are mean ± S.D. of two separate experiments.

The molecular size of cell surface associated HS chains was analyzed by gel chromatography on a Superose 6 column. The elution profiles indicated large differences in HS chain lengths between the different cell lines. MDA-MB-231 produced significantly longer chains and MCF7 much shorter chains than the other cell lines (Figure 3A). Comparing the elution profiles of the labeled HS with the elution positions of size-defined heparin standard polysaccharides and previous calibrations of Superose 6 columns [37], allowed us to estimate the overall size of the HS chains. The peak elution positions of the rather broad peaks correspond to approximately 40–100 kDa for MDA-MB-231, 30–50 kDa for MCF10A, 20–40 kDa for HCC38, and 10–30 kDa MCF7. The very low levels of EXT1 in MCF7 essentially correlated well to the observed shorter chains synthesized by these cells [37,39]. Notably however, although the two triple-negative breast carcinoma cell lines, MDA-MB-231 and HCC38, exhibited almost identical *EXT/L* mRNA profiles their HS chains were of very different lengths, indicating that other factors than the mRNA levels of the EXT family members determine the chain length. Our results furthermore indicated that the reason MCF7 cells have less binding epitopes for the 10E4 antibody might be due to shorter HS chains and thus fewer binding epitopes for the antibody.

HPSE is an endo-β-glucuronidase that cleaves HS side chains of HSPGs [45,46] thereby releasing HS saccharides of different sizes and thus causes truncation of cell surface HS chains. In order to evaluate if the observed differences in HS length could be attributed to HPSE action, we investigated the protein expression of HPSE in MCF10A, MCF7, MDA-MB-231, and HCC38 cells (Figure 3B,C). In particular, MDA-MB-231 cells displayed high levels of the active 50 kDa isoform. The other two tumor cells lines showed intermediate protein expression relative to MDA-MB-231 and MCF10A cells. These findings indicated that the observed differences in chain length were generated during HS synthesis and were not due to HS degradation by endogenously produced HPSE .

### Glycosyltransferase activities

**Figure 4:**
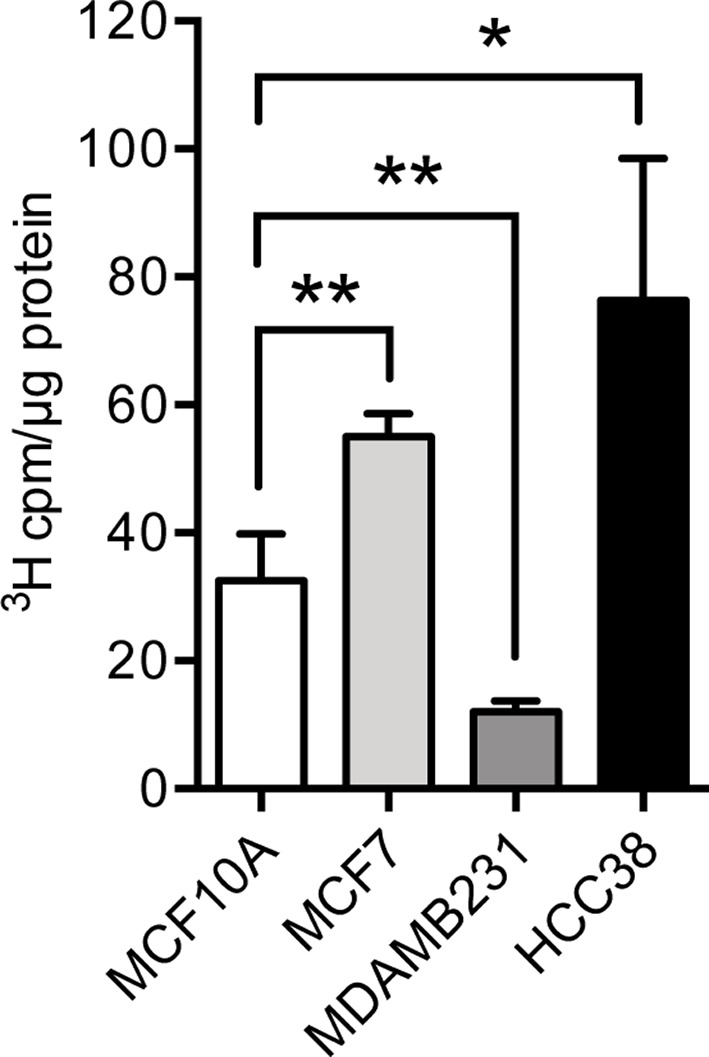
Glycosyltransferase activity Single sugar transfer activities to GlcNAc of crude cell extracts were determined by incubating 10–40 µg lysate protein with 0.125 µCi of UDP-[^3^H]GlcNAc and 45 µg of [GlcA-GlcNAc]*_n_* oligosaccharide acceptors. Labeled oligosaccharide products were isolated by gel chromatography and quantitated by scintillation counting. The error bars represent the mean ± S.D. from three independent experiments, each experiment performed at two different protein concentrations: *P<0.05; **P<0.01.

Lysates of transfected cells were analyzed for GlcNAc-transferase activities with ^3^H-labeled UDP-GlcNAc and a [GlcA-GlcNAc]_*n*_ acceptor as described [37]. The assay essentially measures the activity of the elongation EXT1/EXT2 heterocomplex. The analysis using crude cell lysates clearly demonstrated a large variation in transferase activities between the cell lines. The enzyme activities of HCC38 were approximately two-fold higher than the corresponding values for MCF10 cells*.* Unexpectedly, the enzyme activities of MDA-MB-231 were approximately two-fold lower than the corresponding values for MCF10 cells (Figure 4)*.* In view of these findings and the results from HS chain elongation, it is important to note, however, that the activities reflect activity per milligram of cellular protein. Therefore, the observed differences in activities probably originate from dilution of the EXT proteins in relation to total cellular protein content. Thus, MCF7 cells appeared to contain relatively more EXT1/EXT2 complexes in relation to total cellular protein content.

### Structural characterization of HS

Metabolically labeled HS chains isolated from cell lysate of MCF10A, MCF7, MDA-MB-231, and HCC38 cells were treated with nitrous acid at pH 1.5 (deamination of N-sulphated regions) followed by reduction of the products with NaBH_4_. The reaction mixture was passed through a column of Sephadex G15, and isolated labeled disaccharides were identified by HPLC. The major difference in the relative proportions of sulphated disaccharides was observed in HS isolated from MCF7 cells. MCF7 cells showed a much lower proportion of 2-O sulphated disaccharides and the highest relative proportion of 6-O sulphated disaccharides. These differences were mainly due to a higher proportion of mono-6-O-sulphated disaccharides (Table 3).

**Table 3:** Disaccharide composition of ^35^S-sulphate labeled HS from MCF10A, MCF7, MDA-MB-231, and HCC38 cells

Deamination products	^35^S-labeled deamination products % of total O-sulphated disaccharides			
	MCF10A	MCF7	MDA-MB-231	HCC38
GlcA-aMan_R_6S^1^	9	25.0	2.0	4.0
IdoA-aMan_R_6S	12	15.5	10.5	7.5
IdoA2S-aMan_R_	39	30.5	45.0	43.0
IdoA2S-aMan_R_6S	40	29.0	42.5	45.5
Total%				
2-O-sulphated disaccharides	79	59.5	87.5	88.5
6-O-sulphated disaccharides	61	69.5	55.0	57.0

^35^S-Sulphate labeled samples were degraded to disaccharides (deamination at pH 1.5 followed by reduction of products with NaBH4) that were analyzed by anion-exchange HPLC (see ‘Experimental’ section). Except for MCF10A, the values represent averages of two independent experiments (which varied by ≤3%).

1 aMan_R_, the 2,5-anhydromannitol deamination product of GlcNS residues.

### Extracellularly regulated kinase 1/2 phosphorylation

**Figure 5:**
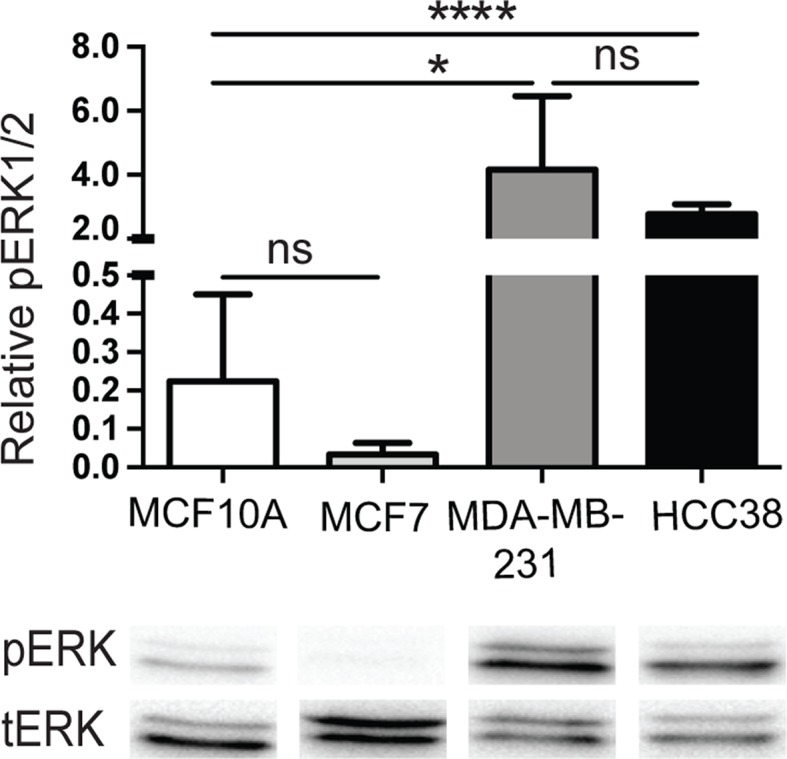
Mitogen-activated protein kinase (MAPK) activity MCF10A, MCF7, MDA-MB-231, and HCC38 cells were serum-starved for 20 h and then cells were lysed. Proteins were separated by SDS/PAGE followed by transfer to nitrocellulose membranes and immunoblotting with an antibody to the phosphorylated forms of ERK1/2 (pERK). After stripping off the nitrocellulose membranes, they were immunoblotted with the non-phosphorylated forms of ERK1/2 (tERK). Shown is one representative result. The histogram shows the quantitation of the ratio of pERK to tERK. The error bars represent the mean ± S.D. from four independent experiments: *P<0.05; ****P<0.0001. Abbreviation: ns, not significantly different.

To determine if the expression levels of the EXTs had a biological effect, we analyzed the activation of the mitogen-activated protein (MAP) kinases, extracellularly regulated kinase (ERK) 1 (ERK1) and ERK2. MCF10A, MCF7, MDA-MB-231, and HCC38 cells were serum starved for 20 h and then analyzed for ERK1/2 phosphorylation by Western blotting. As shown in Figure 5, both MDA-MB-231 and HCC38 displayed high levels of phosphorylated forms of ERK as compared with MCF10A and MCF7 cells. MCF7 cells showed reduced levels of phosphorylation although not significantly different from that of MCF10A cells that displayed a variable degree of p-ERK1/2. These results implied that sustained activation of ERK1/2 appeared to correspond to the malignancy of the breast carcinoma cells.

## Discussion

Although HS biosynthetic enzymes have been reported to be differently regulated in cancer cells, little is known about how these changes in gene expression affect HS structure. In this report, we determined the gene expression levels of the different members of EXT family in the non-tumorigenic MCF10A, the ER-positive MCF7, and the ER, PR, and HER2-triple negative MDA-MB-231 and HCC38 cell lines. In addition, we characterized their HS chains and EXT/L glycosyltransferase activities. The obtained results revealed large variations between the cell lines but also provided evidence that transcriptional activity analyses of HS elongation enzymes are not predictive for HS structure.

The two triple-negative breast carcinoma cell lines exhibited a similar pattern of *EXT* gene expression with significantly increased expression of *EXTL2* and reduced expression of *EXTL3* (Figure 1). This could be a reflection of shared characteristics in the carcinogenic properties of the two cell lines. We were intrigued by the fact that this similarity was not reflected in HS chain length (Figure 3A). Interestingly, the non-tumorigenic MCF10A cells, that like the two triple-negative cell lines have a basal like background, exhibited an HS chain length that was intermediate to that of the MDA-MB-231 and HCC38 cells. The differences in HS chain length appeared to be the result of HS biosynthesis rather than degradation by the HPSE (Figure 3A). One hypothetical explanation to the observed differences in chain length between the MDA-MB-231 and HCC38 cell lines could be differences in the levels and/or types of core proteins. Alternatively, other HS enzymes participating in the biosynthesis machinery of HS may directly or indirectly influence the HS chain elongation.

We have previously shown that a strong reduction in *EXT1* or *EXT2* mRNA levels causes reduced HS chain length, and that increased levels of EXT2, EXTL2, or EXTL3 have little effect on HS chain elongation [38,39]. In agreement with our previous results, MCF7 cells that displayed very low levels of EXT1, synthesized short HS chains. The effect of decreased levels of EXTL3 on HS-chain elongation appears to be cell-type dependent. Reduced levels of *EXTL3* in HEK293 cells results in fewer and longer HS chains [39], whereas knockdown of *EXTL3* in mouse fibroblasts leads to decreased amount of HS, and shorter HS chains [47]. Importantly, the biosynthetic elongation of HS chains appears not only to be dependent on the EXT family of enzymes but also on other enzymes involved in HS biosynthesis and maybe also on unknown modulators of enzyme activity. The HS chain elongation seems to in part be regulated by relatively undefined effects/actions of HS modification enzymes. It has been shown that the cellular levels of the HS modifying enzymes N-deacetylase/N-sulphotransferases (NDSTs) and the HS C5-epimerase influence HS chain elongation. Increased HS chain length has been observed after overexpression of NDST1 or NDST2 [48,49] or HS C5-epimerase [50] in HEK293 cells. In particular, NDST2 has been reported to influence the polymerization of the HS chain [48]. In addition, embryonic stem cells lacking both NDST1 and NDST2, and thus are devoid of N-sulphation, display increased HS chain length without affecting the total amount of HS synthesized by the cells [51]. EXTL3 has been proposed to bind NDST1 and to control its N-sulphotransferase activity [47]. If this association also regulates HS chain length was not investigated. Taken together, the different studies indicate a more complex regulation of chain elongation than merely the activities of the EXT proteins. So far, the significance of HS chain length for carcinogenesis is unclear.

Several investigators have used gene expression profiling approaches of HS enzymes to predict HS structure and to propose tumor biomarkers. Recently, *EXT1* was found to be elevated in plasma of human cholangiocarcinoma patients and proposed to be the prognostic marker for cholangiocarcinoma [52]. Genetic loss of NDST4 in colorectal cancer [53] and high HS 3-O-sulphotransferase 3A expression in HER2-positive breast cancer patients [11] were shown to be associated with poor prognosis. Other studies have investigated how changes in HS biosynthetic enzyme expression levels influence growth factor responses in breast cancer. In a recent publication by Viola et al. [33], they showed that silencing of *EXT1* in MDA-MB-293 cells had surprisingly little effect on cell proliferation, migration, and response to hepatocyte growth factor. In agreement with our results (Figure 5), strong ERK1/2 activation has been demonstrated in serum-starved MDA-MB-231 cells [54]. Our data might suggest that the constitutive activation of ERK1/2 might be correlated to the malignancy of the cells.

Concluding, our data indicated that the malignant status of the breast epithelial cells influenced the mRNA levels of the EXT family, HS sulphation pattern, and the consecutive MAP-kinase activation. The significance of HS chain length in breast carcinoma carcinogenesis remains unclear but HS structure is known to be tissue specific. Importantly, although EXT expression levels correlated with the malignancy of the breast carcinoma cells, the expression levels were not predicative of HS chain length. Therefore, in future studies, structural studies of HS should be considered when considering the impact of transcriptional activity of HS biosynthesis enzymes in cancer.

## Supporting information

**Supplementary figure 1.** EXT/L protein expression

**Supplemental Table:** Primer sequences

## Abbreviations

CS: chondroitin sulphate
DMEM: Dulbecco’s modified Eagle medium
ECM: extracellular matrix
ER: estrogen receptor
EXT: exostosin
EXTL: EXT-like
Erk: extracellularly regulated kinase
HER2: human epidermal growth factor receptor-2
HPSE: heparanase
HS: heparan sulphate
HSPG: HS proteoglycan
MAP: mitogen-activated protein
MO: multiple osteochondroma
NDST: N-deacetylase/N-sulphotransferase
PEST: 1% penicillin and streptomycin
PR: progesterone receptor
qRT-PCR: quantitative real-time PCR
