# Supplementary material for: The exostosin family of glycosyltransferases: mRNA expression profiles and heparan sulphate structure in human breast carcinoma cell lines
Source: Biosci Rep. 2018 Aug 31;38(4):BSR20180770. doi: 10.1042/BSR20180770 (PMC6117623; doi:10.1042/BSR20180770)
Supplement: Supplementary file 1 [file bsr20180770_Supp1.pdf]

Supplemental Table S1. Primer sequences

Human	Forward (5'-3')	Primer position	Reverse (5'-3')	Primer position	Amplicon bp	Sequence ID
EXT1	GCTCTTGTCTCGCCCTTTTGT	814-834	GTGGTGCAAGCCATTCCTAC	917-898	104	NM 000127.2
EXT2	AAGCACCAGGTCTTCGATTACC	1059-1080	GAAGTACGCTTCCCAGAACC	1355-1336	297	NM 000401.3
EXTL1	TCCGAGGTCATCGACTGGAC	1789-1808	AGGAGAAGTAGGCATCCCATA	1936-1916	148	NM 004455.2
EXTL2	ACAGGATGAGAAATCGACTCCA	1040-1061	CTGATGAAGTAGAGACGTGCTTT	1216-1194	177	NM 001439.3
EXTL3	CGCTCATCGCCCACTATTACC	887-907	TGTTCAGCTCTTGGCGCTT	1069-1051	183	NM 001440.3
HPRT1	CTTCCTCCTCCTGAGCAGTC	123-142	TCGAGCAAGACGTTCAGTCC	320-301	198	NM 000194.2
POLR2F	CCCGAAAGATCCCCATCAT	519-537	CACCCCCCAGTCTTCATAGC	583-564	65	NM 001301129.1

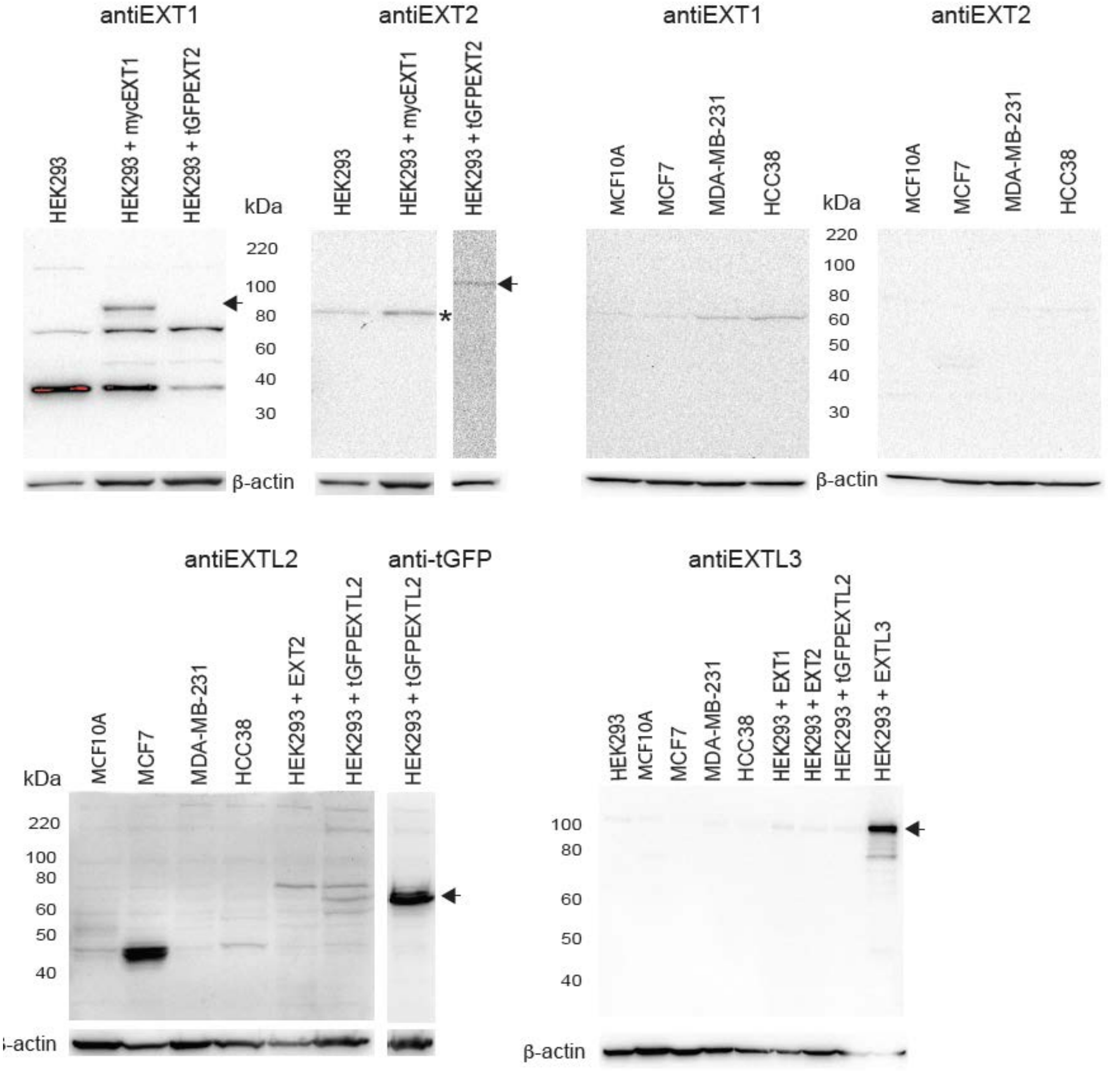

Supplementary figure 1. EXT/L protein expression

Representative immunoblots performed at least three times on cell lysates using the antibodies indicated above the blots. Proteins, approx. 40 µg, were separated on 10% SDS-polyacrylamide gels followed by transfer of proteins to nitrocellulose membranes and immunoblotting with the indicated antibodies. The following EXT antibodies were used; EXT1 (1:1000, mouse monoclonal #sc-515144, Santa Cruz Biotechnology); EXT2 (1:1000, mouse monoclonal #sc-514092, Santa Cruz Biotechnology); EXTL2 (1:1000, rabbit polyclonal #SAB1410351, Sigma-Aldrich) and EXTL3 (1:1000, goat polyclonal #AF2635, R&D-systems. Peroxidase-conjugated secondary goat anti-mouse and goat-anti rabbit IgG were from Santa Cruz Biotechnology. For positive controls EXT-overexpressing cells tagged with turboGFP (tGFP) or myc were used. Myc and tGFP tagged EXT-proteins were detected with anti-myc (1:1000, #087M4765v mouse monoclonal, Sigma-Aldrich) and anti-tGFP (1:1000, #TA150041 mouse monoclonal, ORIGENE), respectively. β-Actin (1:5000, mouse monoclonal #A5441) was from, Sigma-Aldrich. Blots were developed using ECL reagent (Pierce) and the bands visualized with a ChemiDoc XRS imaging system (Bio-Rad).

Note! The star denotes the unspecific 70-75 kDa band in the anti-EXT1 blot was also present in EXT1 deficient cells (data not shown). The arrowheads denote the EXT band.
